# Artificial Intelligence in GI endoscopy: what to expect

**DOI:** 10.3389/fmed.2025.1588873

**Published:** 2025-04-08

**Authors:** María Concepción Aso, Carlos Sostres, Angel Lanas

**Affiliations:** ^1^Digestive Diseases Service, Hospital Clínico Universitario Lozano Blesa, Zaragoza, Spain; ^2^Instituto de Investigación Sanitaria Aragón (IIS Aragón), Zaragoza, Spain; ^3^Department of Medicine, Universidad de Zaragoza, Zaragoza, Spain; ^4^Centro de Investigación Biomédica en Red, Enfermedades Hepáticas y Digestivas (CIBEREHD), Madrid, Spain

**Keywords:** artificial intelligence, endoscopy, colonoscopy, adenoma detection rate, polyp categorization, machine learning, endoscopic navigation, robotization

## 1 Introduction

### 1.1 The evolution of endoscopy and its importance in clinical medicine

From the candle-lit specula used by Hippocrates to visualize the rectum, to Bozzini's illuminated tubes; from the development of semi-flexible endoscopes during the interwar period to the creation of the first fully flexible endoscope by Basil Hirschowitz in the late 1950s, evolution in endoscopy has always been present. In fact, its primary objective remains the same: to achieve a clinical diagnosis with minimal invasiveness, later advancing toward the interventional endoscopy, therefore accomplishing a level of healing previously reserved to surgeons (1, 2).

Nowadays, modern 4K video endoscopes provide exceptional image quality, along with the refinement of scoping materials and tools enable the application of advanced diagnostic and therapeutic techniques, directly impacting on patients care and quality of life.

Merging these already existing technologies with artificial intelligence (AI) represents yet another step in its rapid evolution.

### 1.2 Artificial Intelligence onset in modern medicine. Description of different types of AI in medicine

Artificial intelligence lays in the field of computer science. Its main purpose consists of creating systems with the capacity of accomplishing tasks that would usually require human intelligence, such as voice recognition, problem-solving or decision-making (2).

In medicine, AI made its entry through research, however, it is becoming increasingly present in the day-to-day clinical practice. As for the type of AI mostly used in our area of expertise, the Machine Learning (ML) subfield would be where to draw the attention. Based on the production of different algorithms, it allows the computer to learn from a dataset and enhance its own performance without explicitly programming it to do so. Two primary methodologies are encompassed: supervised learning and unsupervised learning. The former is trained on a labeled datasets, learning progressively from examples provided beforehand. Monitoring this learning and delivering the labeled information is where human scientists and physicians are included. The latter, on the other hand, searches and identifies patterns from raw data, autonomously (3–5).

The algorithms used for Deep Learning are inspired in the human brain functioning. Composed by “nodes” (similar to neurons) that intertwine. These are called Artificial Neuronal Networks (ANN). Regarding image detection and identification, a more efficient type of ANN was developed, known as Convolutional Neural Networks (CNN). The architecture of these networks is based on different layers of nodes and connections, some of them controlled by the developer, and some others considered as “hidden,” in which connections and nodes distribution are unknown. Hidden layers look for prompts, smaller pieces of data or patterns existing in the information given, allowing it to reason and apply that reasoning for future scenarios (3–5).

**Figure 1:**
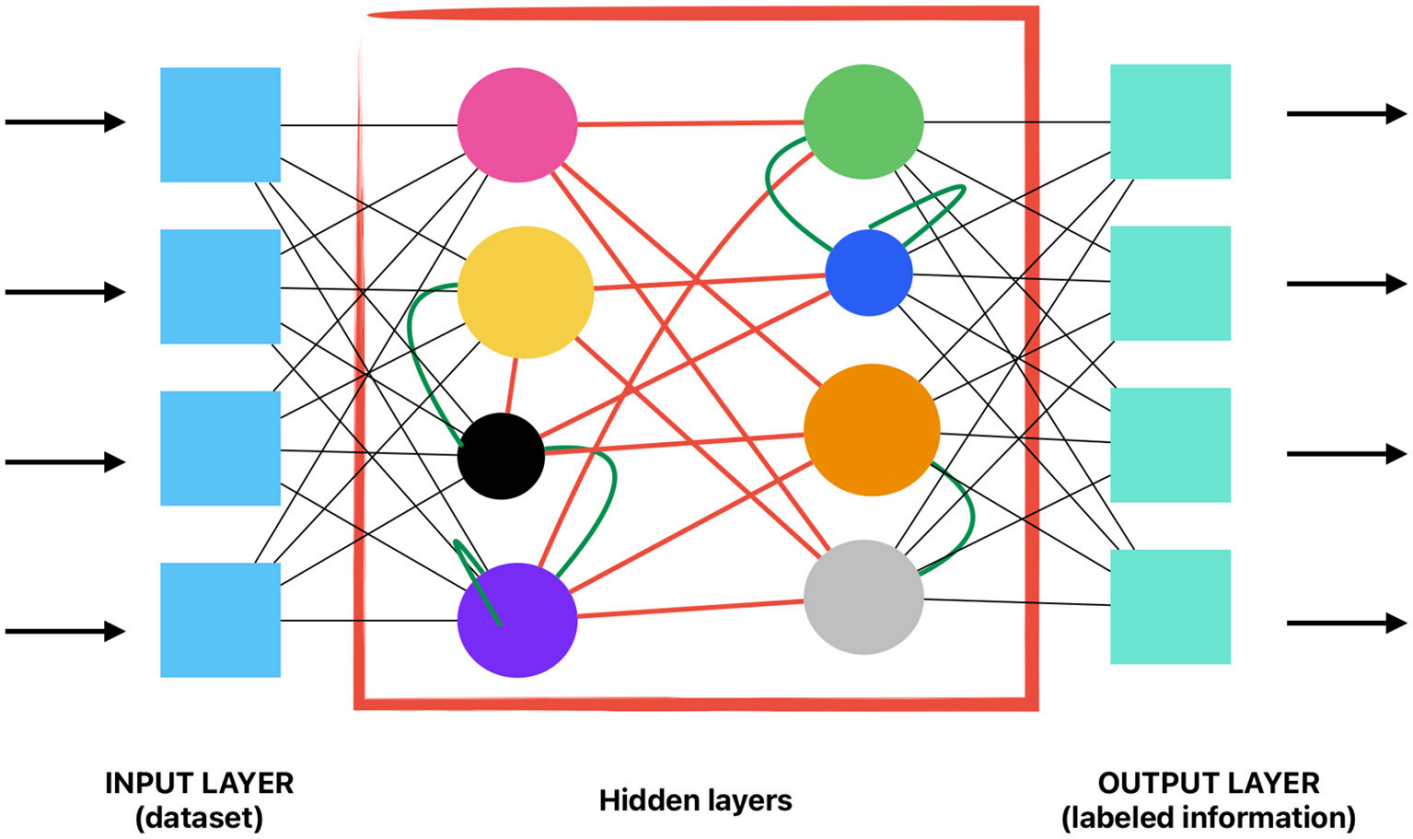
Visual exemplification of a convolutional neuronal network.

Figure 1 aims to simplify the previous information.

### 1.3 Reasons to implement Artificial Intelligence during endoscopic procedures

Convolutional Neural Networks (CNN) can be trained to identify and classify colorectal lesions from an image repository collected from previous colonoscopies. An example on how supervised learning is being applied in colonoscopy could be the identification of polyps in real time during the procedure. For that purpose, the AI has had to be trained beforehand, given multiple images (dataset) in which polyps, or other lesions, could and could not be seen, being told which of the images showcased a polyp and which ones did not (labeling) in a second phase. This information is the one provided by endoscopists or professionals involved and working on AI training.

The detection and potential treatment of premalignant lesions in the digestive track derives in the decrease of cancer incidence. The detection of malignant lesions in early stages improves long-term prognosis or complete remission (6, 7).

Given that colorectal cancer is known to be the second most frequent cause of death according to the Worlds Health Organization (WHO), and the already existing screening programs, optimizing its diagnosis on early stages seems like a logic strategy to invest into. It has been proven that AI-assisted endoscopy directly benefits diagnosis of digestive lesions in their early stages (6, 7).

Endoscopists, both trainees and senior, are fallible, therefore subjected to errors such as missing lesions. It has been demonstrated in many studies that adenoma detection rate (ADR)—a quality indicator broadly considered in endoscopy units worldwide—and precision may vary between physicians and it is enhanced through AI, especially when tackling flat or millimetric lesions and sessile serrated lesions. Fatigue also plays a significant role in the quality of the endoscopy, as well as the expertise of the operator. Various meta-analyses already compare performance during standard vs. AI-assisted colonoscopies for novel endoscopists. Publications of Yamaguichi and Yao evaluated the results of 7 randomized controlled trials (RCTs) in total, showing that adenoma detection rate (ADR) and adenoma miss rate (AMR) among other secondary metrics, where significantly improved in the AI-assisted branch, thus improving overall outcomes for trainees. Not only that but also remarking AI's role as an enhancer of trainees learning process (6, 8, 9).

However, not all studies point out in that direction, since other studies show a minimal sensibility improvement on AI-assisted endoscopies at the cost of sacrificing an increase in total procedure time and workload, without translating into a significant impact on follow-up recommendations. These findings cast doubt on whether AI-assisted endoscopy will become the indispensable tool it is said to be (10).

The role of AI in endoscopy should not be seen as a substitution of the physician, but rather the creation of a useful aid to assist the physician during the procedure, improving not only adenoma detection rate (ADR) and adenoma miss rate (AMR) but also providing information on the polyp's nature itself (potentially malignant or benign), thus enabling the endoscopist to better lay out the resection approach. In fact, some studies have delved deeper into the relationship between AI as a tool and the endoscopist, evaluating different scenarios, such as the option for the endoscopist to decide when to request AI assistance. The results of Zander et al. seem to indicate an improvement in the endoscopist's confidence in AI in these mixed models (CADx). With the improvement of the endoscopist's confidence in AI-assisted diagnosis, new polyp management options emerge, such as the “resect and discard” strategy, in which the polyp is removed but not retrieved since the pre-test characteristics are benign, or the “diagnose and leave behind” strategy for patients in whom a lesion categorized as benign is detected and who have a high risk of bleeding (6, 11).

## 2 Material and methods: search strategy. Inclusion and exclusion criteria

Information was gathered from Pubmed. Preliminary data structuring and keyword selection was assisted by AI generators. Diagrams were created using Freeform.

Out of 9,576 articles, a narrower selection was performed using the following criteria:

Inclusion criteria: papers published in 2018 or after, including narrative reviews.
Exclusion criteria: papers published before 2018 were excluded. Papers written in other languages different from English or Spanish were excluded. General documents and case reports were excluded.

Specific information on AI and ML was searched on GoogleScholar.

A narrative analysis and review of the articles was later conducted in order to write this paper.

## 3 Discussion

### 3.1 Update advances: IA and adenoma detection rate and polyp classification

As already mentioned, ML algorithms, more specifically CNNs can be trained to detect and classify polyps (and potentially other lesions in the near future) during the endoscopy, by processing the images in real time.

After a sufficient training, AI is able to highlight areas in which a polyp is suspected to be, sometimes not that evident to the human eye, allowing the endoscopist to inspect that area in more depth (6, 9). Following that line, the next step to delve into was not only detection—and the decrease of false positives alongside—but also diagnosis.

By estimating the pre-resection probability of the polyp's histological nature, the endoscopist can determine the most appropriate resection technique. In cases where malignant degeneration without deep submucosal invasion is suspected, a wider-margin resection or even a submucosal dissection may be considered. Conversely, for lesions classified as benign and in selected cases—such as elderly patients with a high risk of bleeding—future approaches may explore surveillance without resection as a viable alternative, since the better characterization of benign lesions could allow the endoscopist to leave benign lesions behind.

The training of these estimations is done by labeling polyps according to post resection pathology results, labeling done by pathologists in this case. Here is where the hidden layers in the CNN look for prompts and connections unknown to humans and, if correct, to carry out a task impossible for the endoscopist at that moment, providing an estimation of pathology results before resection (3, 4, 11).

Currently, various AI-assisted systems are available for both polyp detection and classification (see Table 1) (5, 12). These systems commonly use visual markers, such as boxes or circles, to highlight areas of interest. For polyp characterization, most rely on a color-coded classification scheme—typically red, yellow, and green—supplemented by additional data displayed on the screen.

**Table 1:** AI software programs currently in use.

**Principal AI softwares in use**			
**Name**	**Developer**	**Functions**	**Considerations**
GI Genius	Medtronic	Polyp detection in real time	Europe approved. Ongoing studies.
EndoBrain and EndoBrain-EYE	Olympus	Polyp detection in real time and classification/potential malignancy	Uses NBI and magnification. Europe approved. Ongoing studies.
CAD-EYE	Fujifilm	Polyp detection in real time and classification/potential malignancy	Uses BLI/LCD. Europe approved. Ongoing studies.
Discovery	Pentax	Polyp detection in real time	Europe approved. Ongoing studies.
CADDIE	Odin Vision	Polyp detection in real time and classification/potential malignancy	Finished EAGLE trial. Preprint

#### 3.1.1 Other areas of development: Navigation softwares

As previously mentioned, most current AI research focuses on automatic detection of polyps or polyp classification, but the exact location of the scope and its navigation through the colon relies entirely on the endoscopist's clinical experience. Nonetheless, several lines of investigation are headed toward the creation of navigation software programs.

It is broadly known that the variability between patients' anatomies becomes a challenge when trying to stay oriented inside the colon, specially at the beginning of endoscopy training. The length of the scope or the characteristics of the mucosa are not always reliable.

The efforts are concentrated on automate localization within the colon using technologies like Simultaneous Localization And Mapping (SLAM), an AI computational approach to constructing or updating a topological map of an unknown environment while simultaneously keeping track of the scope location within it. These maps could potentially be used to re-localize a location spotted on the map, for example a polyp during a second exploration, determine the percentage of colonic mucosa correctly visualized and even applications in polyp size measurements (13–15).

The map is a graph composed of nodes that represent distinctive sections of the colon anatomy, and edges that link places that are connected in space. Localization is based on image similarity by means of a deep learning descriptor (13–15).

One of the limitations of this technology under development is the need for a previous complete endoscopy and processing of data, not being able to use de navigation system during the initial colonoscopy yet. This is an area to improve in the foreseeable future, for example during withdrawal of the scope using data collected during insertion. Another aspect that remains unresolved is the variability in lighting within the colon, as well as the deformation of tissues, which has presented challenges for the analysis of images by the algorithm (15).

Even so, progress continues to be made and it is undeniable that thanks to this technology we might being witnessing the first steps toward robotic endoscopy.

### 3.2 Challenges and limitations

Numerous challenges and limitations facing AI have already been acknowledged, many of which are exacerbated by the rapid pace at which advancements are being made.

On one hand, there are technical limitations. Algorithms are designed to perform a wide range of tasks; however, when it comes to implementing AI in endoscopy, it has become evident that the development of compatible and functional hardware is slower and more costly. This is clearly exemplified in the case of robotics, due to the high financial costs involved (16, 17).

On the other hand, there are social challenges to be confronted. The astonishing speed at which AI advancements are occurring has not allowed sufficient time to adapt and regulate its use responsibly. This applies in terms of legal frameworks, ethical considerations and the reliability of the responses provided by AI systems.

The European Union has been working on multiple regulations on this matter, with the dual purpose of ensuring AI development and excellence while protecting users from related threats, particularly in terms of privacy and reliability. Initiatives such as Digital Europe and Horizon Europe aim to support the development of AI, with similar efforts taking place across the world. Through the regulatory framework and the “AI Act,” policies are being designed to ensure the transparency and reliability of the technology, as well as to address the responsibilities arising from improper or unreliable use, many of which fall on the developing company (18, 19).

## 4 Future perspectives

### 4.1 Artificial Intelligence developments: endoscopic capsule, inflammatory bowel disease

Some groups are advancing rapidly in AI assisted upper endoscopy. For example Fockens et al. or Jukema et al. recently published studies in which a significant improvement was found in both sensibility and specificity for Barrett lesion detection. Although the sample size for the Fockens study was small−30 patients- (7, 20), these results seem optimistic for the future of AI in upper endoscopy (2, 5, 21).

A capsule endoscopy model focused on the optical diagnosis of colonic lesions is being developed. This model integrates AI for image analysis and interpretation, supported, like many other innovations, within the framework of the European project HORIZON 2.0. The primary aim of this line of research focuses on optimizing the sensitivity of diagnosis by minimizing physicians' workload and reducing the risks associated with more invasive procedures, such as colonoscopies and enteroscopies. Although it does not offer therapeutic approaches, it enables patient screening without requiring time investment in image interpretation (22).

As for the advances in IBD, a systematic review on the topic (23) concludes that AI-assisted endoscopy could help the diagnostic process of IBD in the near future, addressing important matters such as grading mucosal inflammation, as well as differentiating between ulcerative colitis (UC), Crohn's disease (CD) or other pathologies presenting with mucosal inflammation such as ischemic or infectious. Dysplasia detection in IBD patients was also addressed.

In relation to advanced endoscopy, AI will also play its part assisting ERCP (Endoscopic retrograde cholangiopancreatography) and EUS (Endoscopic UltraSound). He et al. are trying to develop a model in which AI helping the characterization of submucosal lesions through EUS (21, 24).

### 4.2 Robotic endoscopy

Three types of devices are available nowadays in order to obtain images from the inside of the gastrointestinal (GI) tract: rigid endoscopes (mainly used in NOTES (Natural Orifice Translumenal Endoscopic Surgery) or TAMIS (TransAnal Minimal Invasive Surgery), flexible endoscopes, and endoscopic capsules.

The robotization of these equipment is one of the fields of most intense research, given the recent advancements in the endoscopic integration of AI, particularly in AI-assisted navigation systems. In this regard, two concepts emerge (16, 17):

– Robot-assisted endoscopy, where a robot aids in supporting and managing the scope.
– Active gastrointestinal endoscopy, where the robotic device includes propulsion or remote control systems for potentially autonomous movement within the GI tract, either through magnets, anchoring and advancing systems, or wheel and crawler-based systems. More specifically, in the field of endoscopic capsules, models with propulsion motors, fins, and propellers are being developed, mimicking designs already used in other scientific areas such as aeronautics as well as magnet-based control systems, similar to those proposed for colonoscopy.

Several prototypes and trials worldwide already show promising results over conventional and AI-assisted endoscopy alone, such as improved ergonomics and one-hand control capability, an increase on image stability, distance and pressure feedback to prevent perforations, and enhanced stability of the endoscope during therapeutic procedures, all of it AI-enhanced.

The EndoMaster device, which is being developed by Dr. Chiu's team, can be taken as an example. They have recently published a prospective, single-arm phase II clinical trial on its performance and safety in endoscopic submucosal dissection (ESD) demonstrating results that support the strengths of endoscopic robotization (25).

Assistance for trainee endoscopists is also one of its key strengths, as it improves precision and minimizes risks. Additionally, it promises benefits in reducing endoscopist fatigue, as well as incorporating more sophisticated surgical tools and multi-port approaches with triangulation, opening the door to techniques similar to those developed for laparoscopic or NOTES surgery (16, 17).

However, challenges need to be tackled before these technologies can become a routine in clinical practice. The most notable ones being the high cost of the devices and the large size of current prototypes thus making them more rigid and harder to reach challenging areas such as the cecum. Despite these challenges, robotization is emerging as the most likely future for endoscopy, understood as a robot-human partnership where the role of the endoscopist will continue to play a central part throughout patient care (16, 17).

### 4.3 Modifications to expect in clinical practice. Precision medicine and prevention

Many authors argue that the implementation of AI in medicine is set to revolutionize chronic disease management. AI, through deep learning and CNNs, is expected to analyze big data and be able to identify patterns. Combined with lifestyle information, analytical parameters, and chronic medication data, AI could integrate global knowledge about a specific patient and predict risk factors for disease development with superior accuracy.

AI-assisted basic endoscopy is already a reality. Using live images during a routine endoscopy, AI could potentially predict the risk of bleeding recurrence when first evaluating a gastric ulcer or maybe predict the risk of colorectal cancer development in the absence of mucosal lesions. Nonetheless, challenges remain, including legal concerns and the difficulty of training models with such vast data (26, 27).

## 5 Conclusion

Although AI presents challenges, we strongly believe its implementation has the potential to revolutionize endoscopy, improving clinical outcomes and patient care. A collective effort needs to be made in order to facilitate its growth, both scientifically and in daily practice. More meta-analyses will be needed in the future due to the exponential output in this field, along with an increase of video-based trials (as opposed to still frames) to validate current technology. Regulations regarding its use, not only at the European level but also globally, will need to be established in the coming years. This will require a joint effort from the scientific community along with governments to ensure the responsible use of this technology, always placing the wellbeing and benefit of the patient at the center of all decisions.

## References

[1] Herrera-Esquivel JJ Patiño-Suárez K Délano-Alonso R. Evolución de la endoscopía y la cirugía endo/laparoscópica; pasado, presente y futuro. Rev Mex Cir Endoscop. (2018) 19:131–136.

[2] Kaul V Enslin S Gross SA. History of artificial intelligence in medicine. Gastrointest Endosc. (2020) 92:807–12. 10.1016/j.gie.2020.06.040 32565184

[3] He K Zhang X Ren S Sun J. Deep residual learning for image recognition. In: Proceedings of the IEEE Conference on Computer Vision and Pattern Recognition (CVPR). Las Vegas, NV, USA: IEEE (2016). p. 770–778. 10.1109/CVPR.2016.90

[4] He K Zhang X Ren S Sun J. Identity mappings in deep residual networks. In: Leibe B, Matas J, Sebe N, Welling M, editors. Computer Vision – ECCV 2016. Cham: Springer (2016). p. 630–645. 10.1007/978-3-319-46493-0_38

[5] Okagawa Y Abe S Yamada M Oda I Saito Y. Artificial intelligence in endoscopy. Dig Dis Sci. (2022) 67:1553–72. 10.1007/s10620-021-07086-z 34155567

[6] Jin X-F Ma H-Y Shi J-W Cai J-T. Efficacy of artificial intelligence in reducing miss rates of GI adenomas, polyps, and sessile serrated lesions: a meta-analysis of randomized controlled trials. Gastrointest Endosc. (2024) 99:667–75.e1. 10.1016/j.gie.2024.01.004 38184117

[7] Fockens KN Jukema JB Jong MR Boers T van der Putten JA Kusters CHJ. The use of a real-time computer-aided detection system for visible lesions in Barrett's esophagus during live endoscopic procedures: a pilot study (with video). Gastrointest Endosc. (2024) 100:527–31. 10.1016/j.gie.2024.04.011 38604297

[8] Yao L Li X Wu Z Wang J Luo C Chen B. Effect of artificial intelligence on novice-performed colonoscopy: a multicenter randomized controlled tandem study. Gastrointest Endosc. (2024) 99:91–9.e9. 10.1016/j.gie.2023.07.044 37536635

[9] Yamaguchi D Shimoda R Miyahara K Yukimoto T Sakata Y Takamori A. Impact of an artificial intelligence-aided endoscopic diagnosis system on improving endoscopy quality for trainees in colonoscopy: prospective, randomized, multicenter study. Dig Endosc. (2024) 36:40–8. 10.1111/den.14573 37079002 PMC12136242

[10] Sinonquel P Eelbode T Pech O De Wulf D Dewint P Neumann H. Clinical consequences of computer-aided colorectal polyp detection. Gut. (2024) 73:1974–80. 10.1136/gutjnl-2024-331943 38876773

[11] van der Zander QEW Roumans R Kusters CHJ Dehghani N Masclee AAM de With PHN. Appropriate trust in artificial intelligence for the optical diagnosis of colorectal polyps: the role of human/artificial intelligence interaction. Gastrointest Endosc. (2024) 100:1070–1078.e10. 10.1016/j.gie.2024.06.029 38942330

[12] Kader R Hassan C Lanas Á Romańczyk M Romańczyk T Kotowski B. International multi-centre randomised controlled trial of a novel cloud-based artificial intelligence polyp detection system (CADDIE) - EAGLE Trial [Preprint]. Available online at: ClinicalTrials.gov (NCT05730192) (accessed February 16, 2025).

[13] Munguía-Alcalá RF Grau-Saldes A SLAM. con mediciones angulares: método por triangulación estocástica. Ingenierí*a Investigación y Tecnol*. (2023) 14:257–74. 10.1016/S1405-7743(13)72241-8

[14] Sostres C Aso MC López de la Cruz J Azagra P Recasens D Morlana J. ENDOMAPPER: SLAM visual en colonoscopia. Rev Gastroenterol Hepatol. (2023) 46:7. 10.1016/S0210-5705(23)00139-5

[15] Azagra P Sostres C Ferrández Á Riazuelo L Tomasini C Barbed OL. Endomapper dataset of complete calibrated endoscopy procedures. Sci Data. (2023) 10:671. 10.1038/s41597-023-02564-7 37789003 PMC10547713

[16] Cui Y Thompson CC Chiu PWY Gross SA. Robotics in therapeutic endoscopy (with video). Gastrointest Endosc. (2022) 96:402–10. 10.1016/j.gie.2022.05.019 35667390

[17] Li Z Chiu PWY. Will the robot take over endoscopy? Endoscopy. (2015) 47:773–4. 10.1055/s-0034-1392420 26317584

[18] European Commission. European approach to artificial intelligence. Brussels: European Commission. (2021). Available online at: https://digital-strategy.ec.europa.eu/en/policies/european-approach-artificial-intelligence (accessed February 21, 2025).

[19] Aung YYM Wong DCS Ting DSW. The promise of artificial intelligence: a review of the opportunities and challenges of artificial intelligence in healthcare. Br Med Bull. (2021) 139:4–15. 10.1093/bmb/ldab016 34405854

[20] Jukema JB Kusters CHJ Jong MR. Computer-aided diagnosis improves characterization of Barrett's neoplasia by general endoscopists. Gastrointest Endosc. (2024) 100:616–25.e8. 10.1016/j.gie.2024.04.013 38636819

[21] Penrice DD Rattan P Simonetto DA. Artificial intelligence and the future of gastroenterology and hepatology. Gastro Hep Adv. (2022) 1:581–95. 10.1016/j.gastha.2022.02.025 39132066 PMC11307848

[22] AI-supported picture analysis in large bowel camera capsule endoscopy (AICE). Grant agreement ID: 101057400. Horizon Europe - Health. European Commission (2022–2026). Available online at: https://cordis.europa.eu/project/id/101057400 (accessed February 26, 2025).

[23] Pal P Pooja K Nabi Z Gupta R Tandan M Rao GV. Artificial intelligence in endoscopy related to inflammatory bowel disease: a systematic review. Indian J Gastroenterol. (2024) 43:172–87. 10.1007/s12664-024-01531-3 38418774

[24] Cai M Song B Deng Y Gao P Cai S Yalikong A. Automatically optimized radiomics modeling system for small gastric submucosal tumor (< 2 cm) discrimination based on EUS images. Gastrointest Endosc. (2024) 99:537–47.e4. 10.1016/j.gie.2023.11.006 37956896

[25] Chiu PWY Yip HC Chu S Chan SM Lau HSL Tang RSY. Prospective single-arm trial on feasibility and safety of an endoscopic robotic system for colonic endoscopic submucosal dissection. Endoscopy. (2025) 57:240–6. 10.1055/a-2411-0892 39242090 PMC11867098

[26] Subramanian M Wojtusciszyn A Favre L Boughorbel S Shan J Letaief KB. Precision medicine in the era of artificial intelligence: implications in chronic disease management. J Transl Med. (2020) 18:472. 10.1186/s12967-020-02658-5 33298113 PMC7725219

[27] Johnson KB Wei WQ Weeraratne D Frisse ME Misulis K Rhee K. Artificial intelligence in gastroenterology. Clin Transl Sci. (2021) 14:86–93. 10.1111/cts.12884 32961010 PMC7877825

